# Remote scoring models of rigidity and postural stability of Parkinson’s disease based on indirect motions and a low-cost RGB algorithm

**DOI:** 10.3389/fnagi.2023.1034376

**Published:** 2023-02-17

**Authors:** Ling-Yan Ma, Wei-Kun Shi, Cheng Chen, Zhan Wang, Xue-Mei Wang, Jia-Ning Jin, Lu Chen, Kang Ren, Zhong-Lue Chen, Yun Ling, Tao Feng

**Affiliations:** ^1^Center for Movement Disorders, Department of Neurology, Beijing Tiantan Hospital, Capital Medical University, Beijing, China; ^2^China National Clinical Research Center for Neurological Diseases, Beijing, China; ^3^GYENNO SCIENCE CO., LTD., Shenzhen, China; ^4^HUST-GYENNO CNS Intelligent Digital Medicine Technology Center, Wuhan, China; ^5^Department of Encephalopathy I, Dong Fang Hospital Affiliated to Beijing University of Chinese Medicine, Beijing, China; ^6^Parkinson's Disease Center, Beijing Institute for Brain Disorders, Beijing, China

**Keywords:** Parkinson’s disease, remote, computer vision, machine learning, rigidity, postural stability

## Abstract

**Background and objectives:**

The Movement Disorder Society’s Unified Parkinson’s Disease Rating Scale Part III (MDS-UPDRS III) is mostly common used for assessing the motor symptoms of Parkinson’s disease (PD). In remote circumstances, vision-based techniques have many strengths over wearable sensors. However, rigidity (item 3.3) and postural stability (item 3.12) in the MDS-UPDRS III cannot be assessed remotely since participants need to be touched by a trained examiner during testing. We developed the four scoring models of rigidity of the neck, rigidity of the lower extremities, rigidity of the upper extremities, and postural stability based on features extracted from other available and touchless motions.

**Methods:**

The red, green, and blue (RGB) computer vision algorithm and machine learning were combined with other available motions from the MDS-UPDRS III evaluation. A total of 104 patients with PD were split into a train set (89 individuals) and a test set (15 individuals). The light gradient boosting machine (LightGBM) multiclassification model was trained. Weighted kappa (*k*), absolute accuracy (*ACC ± 0*), and Spearman’s correlation coefficient (*rho*) were used to evaluate the performance of model.

**Results:**

For model of rigidity of the upper extremities, *k* = 0.58 (moderate), *ACC ± 0* = 0.73, and *rho* = 0.64 (moderate). For model of rigidity of the lower extremities, *k* = 0.66 (substantial), *ACC ± 0* = 0.70, and *rho* = 0.76 (strong). For model of rigidity of the neck, *k* = 0.60 (moderate), *ACC ± 0* = 0.73, and *rho* = 0.60 (moderate). For model of postural stability, *k* = 0.66 (substantial), *ACC ± 0* = 0.73, and *rho* = 0.68 (moderate).

**Conclusion:**

Our study can be meaningful for remote assessments, especially when people have to maintain social distance, e.g., in situations such as the coronavirus disease-2019 (COVID-19) pandemic.

## 1. Introduction

Parkinson’s disease (PD) is the second most common neurodegenerative disorder and is characterized by a broad spectrum of gradually developing motor and non-motor impairments (Selikhova et al., 2009). At present, in clinical practice, measurement of the various aspects of PD and their severity relies mostly on clinically based rating scales, as no specific biomarker or imaging index can evaluate PD symptoms as a whole thus far. The Movement Disorder Society’s Unified Parkinson’s Disease Rating Scale (MDS-UPDRS), comprising four sections, is the scale most commonly used to evaluate global severity of PD, among which Part III is applied to assess motor symptoms in detail (Goetz et al., 2007, 2008).

As PD is a chronic progressive disease, long-term follow-up is essential to evaluate severity and adjust drug regimens for patients. However, during the coronavirus disease-2019 (COVID-19) pandemic, follow-up on-site clinic visits have been a problem since clinical stability and infection prevention are difficult to simultaneously guarantee (Goetz et al., 2020). Under circumstances such as COVID-19, telemedicine and digital visits have become more important for ensuring the quality of healthcare and safe distancing (Prasad et al., 2020). A review related to the application of artificial intelligence in PD mentioned that instrumentations from previous studies, including camera systems, inertial measurement unit sensors, and electromyography sensor tracking, were used to build machine learning models for obtaining MDS-UPDRS III scores (Belic et al., 2019). Compared with other wearable sensors, the most available pattern during COVID-19 was using a vision algorithm remotely, since participants did not need to be trained in wearing the sensors and ensuring the accuracy of the process. Moreover, vision-based remote assessment can be a time-saving, resource-saving, well-accepted tool for both patients and doctors (Xu et al., 2021). The red, green, and blue (RGB) color model is one of the most low-cost and widely available methods for online follow-ups since it can be applied through most smartphone cameras.

The MDS-UPDRS III evaluates multiple dimensions of motor dysfunction, including speech, facial expression, tremor, rigidity, bradykinesia, posture, and gait (Goetz et al., 2007). Several motions related to tremor, bradykinesia, and axial symptoms have been studied for scoring based on vision instrumentation. Kye Won Park et al. built two models for scoring resting tremor and finger tapping by using OpenPose and video clips, respectively (Park et al., 2021). Lu et al. (2020) proposed a vision-based deep learning model for assessing the severity of gait and posture. However, rigidity can be impossible to achieve by vision, and posture stability can be unsafe without trained examiner remotely, because scoring rigidity requires an examiner to touch the patient, and scoring postural reflexes requires a trained health examiner to ensure safety during the whole process of pullback test (Goetz et al., 2015). More importantly, if six values from the MDS-UPDRS III assessment, including rigidity of the neck (Rig-Neck), lower extremities (Rig-LE), and upper extremities (Rig-UE) and postural stability (PS), are lost, the total score will not be valid and would lie outside the permissible threshold (Goetz et al., 2015). Many previous studies have found that features extracted from other motions were correlated with rigidity. Rigidity in PD was found to be associated with the reduction in leg and arm swing during gait assessment (Kwon et al., 2014) and the speed of the release of the keyboard during the finger-tapping motion on an engineered keyboard (Trager et al., 2020). Regarding PS in PD, Claudia Ferraris et al. estimated PS by extracting features from a quiet stance (Ferraris et al., 2019). Therefore, these motions can be potential predictors for evaluating rigidity and PS scores. We examined three types of features, including position signal features, angle features, and kinematic features, based on previous studies and their impact on MDS-UPDRS III rigidity scores.

Based on the mentioned hypothesis and methods, our study used a machine learning-based system with an RGB camera and features extracted from the available motions to estimate rigidity and PS in patients with PD. These motions are safe for the patient to complete independently or under the supervision of caregivers. This system could solve the problem of estimating rigidity and PS in a home-based environment, making it possible to determine the whole MDS-UPDRS III score together with other evaluations by vision, especially during situations such as the COVID-19 pandemic.

## 2. Materials and methods

### 2.1. Protocol

In this study, 104 patients with PD were enrolled at Beijing Tiantan Hospital, Capital Medical University, from 1 March 2020 to 31 December 2021. All patients were diagnosed according to the 2015 MDS PD criteria (Postuma et al., 2015). Written informed consent was obtained from all individuals. The study was approved by the ethics committees of Beijing Tiantan Hospital. Demographic information (e.g., age, gender, and disease duration) was collected. All patients were asked to complete all the motions of the MDS-UPDRS III in front of the camera. All MDS-UPDRS III scores were assessed by two specialists in movement disorders (LY M and HZ M), both of whom had passed the MDS-UPDRS training program, and kappa consistency test was performed. The kappa value (measured by kappa consistency test) between the raters was 0.93. Ture labels of PS and rigidity (neck, two upper extremities, and two lower extremities) were collected by them on-site.

Only patients with completed videos were selected. Because of the device, some videos had some problems of blur, which can cause the disidentification of joints. Therefore, originally the whole sample size was 108, but 104 patients had all of completed 11 motion videos.

Stratified sampling based on the total score of rigidity and postural stability was used to split train and test set randomly. For making sure they are balanced, Chi-square test, T-test, and Wilcoxon test were used to test the difference of sex, normally distributed data, and non-normally distributed data, between the two groups. Sex was described as number and percentage. Normally distributed data were described as mean and SD. Non-normally distributed data were described as median and interquartile range (25% quantile and 75% quantile).

### 2.2. Data acquisition

In this study, Microsoft Kinect V2 was used to film all the evaluation RGB videos. The data from the facial expression video were 1,080 p @20fps. Data from the other videos were 540p @20fps. Our study was from a project that decided to build up a system for evaluating every item of MDS-UPDRSIII completely by vision. Therefore, we selected motions in the MDS-UPDRSIII, these motions not only can be source for evaluating of their corresponding items, but also can be source for evaluating rigidity and PS indirectly. There were 11 motions, including “facial expression” (FE), “finger tapping” (FT), “hand movements” (HM), “pronation-supination movements of hands” (PSOH), “toe tapping” (TT), “leg agility” (LA), “arising from chair” (AFC), “GAIT,” “posture” (POS), “postural tremor of hands” (PTOH), and “kinetic tremor of hands” (KTOH). Except for pullback test of PS, and rigidity, all these 11 motions were available in video-based circumstance (Goetz et al., 2020).

### 2.3. Evidence before this study

Before designing the feature engineering, we reviewed and studied some results from past studies about the relationships between particular motions and either rigidity or PS. In addition, we considered the specific muscle or joints that typically move during the evaluation of rigidity and PS for the MDS-UPDRS III. During the rigidity evaluation, the examiner tested the passive movements of the major joints and neck of the participant in conditions with or without an activation maneuver, including FT and TT (Goetz et al., 2008). These joints included the wrist, elbow, hip, and knee. The motions of these joints can also be found in other motions. Specifically, during the motion used to assess LA, the participant is asked to raise their foot and then stomp down on the ground. The knee of the participant is involved during the whole process. Information related to the ankle or joint could be used to represent the severity of rigidity. Regarding the reviewed studies, we found that three types of motions with different patterns could be extracted for this study. One type was based on the position signal during joint motion. Shan et al. (2001) found that reductions in rigidity were correlated with angular excursions of the ankle during the GAIT test when analyzing the effects of levodopa and tolcapone (Spearman’s correlation coefficient = −0.46, value of *p* < 0.001). The second type was based on evaluating the range of motion (ROM) in the ankles and trunk during different motions. Roberto Cano-de-la-Cuerda found that rigidity of the trunk extensor muscles was related to trunk flexion and extension ROM (Spearman’s correlation coefficient = −0.534, value of *p* = 0.042) based on their research analyzing functional mobility and quality of life (Cano-de-la-Cuerda et al., 2020). The third type was based on kinematic characteristics, including speed and amplitude. A study by Megan H Trager indicated that the speed of key release on a keyboard during a FT motion was related to rigidity in the upper extremity (Pearson’s correlation coefficient = −0.58; value of *p* < 0.01; Trager et al., 2020).

### 2.4. Feature engineering algorithm

**Figure 1:**
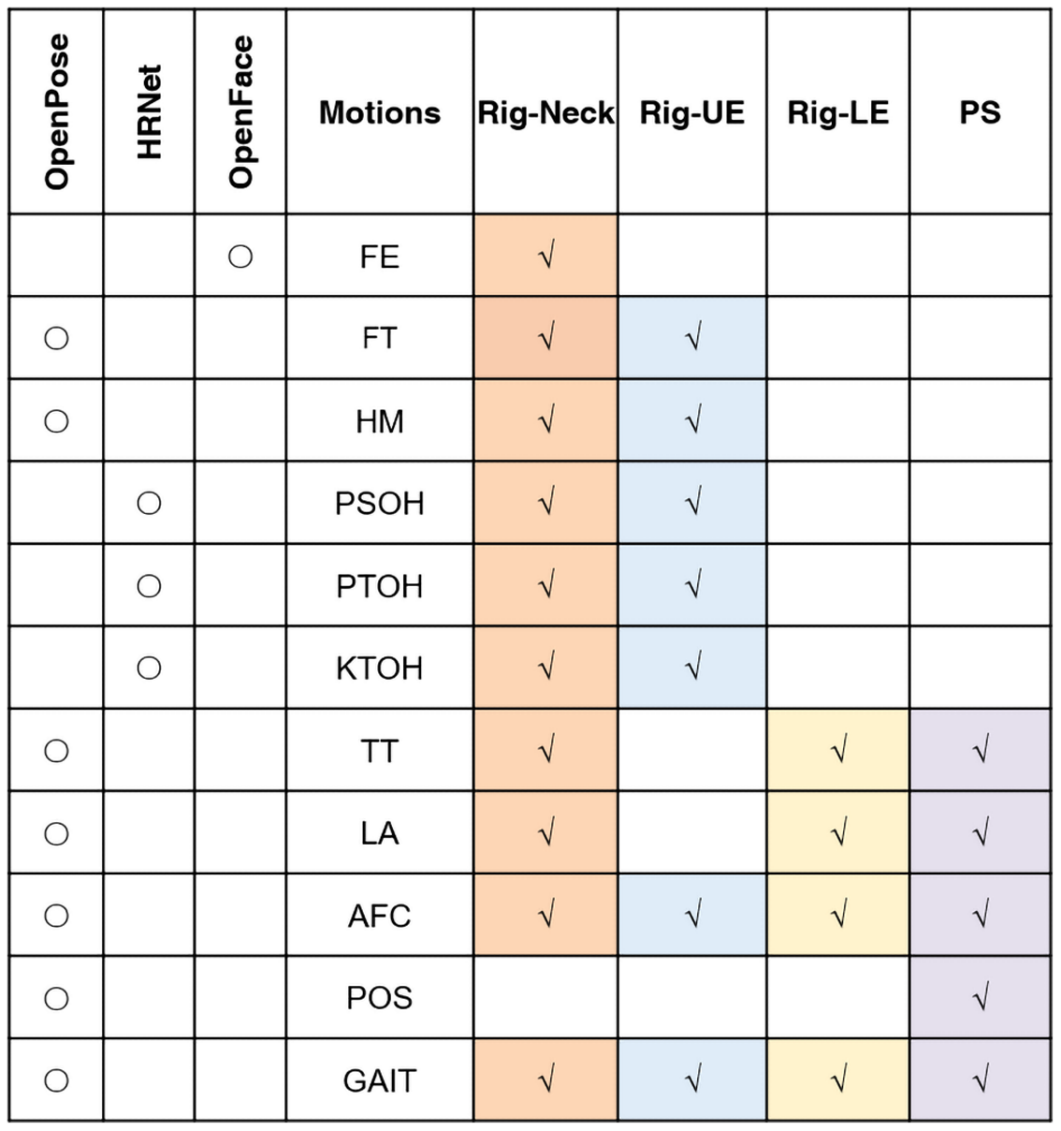
Relationships between movements, techniques and models. Rig, rigidity; UE, upper extremity; LE, lower extremity; PS, postural stability; AFC: arising from chair; FE, facial expression; FT, finger tapping; HM, hand movements; KTOH, kinetic tremor of the hands; LA, leg agility; POS, posture; PSOH, pronation/supination of the hands; PTOH, postural tremor of the hands; TT, toe tapping; AFC: arising from chair; and FE, facial expression.

This study included 11 motions. Figure 1 shows the relation between the different motions and the techniques we used. Different methods were used for different motions based on the characteristics of these motions. OpenFace (Baltrušaitis et al., 2016) was used for extracting features of FE. The facial-action-coding system defines the correspondence between facial emotions and facial muscles and divides facial expressions into 46 action units (AU). OpenPose (Cao et al., 2021) was used for pose estimation, which can provide estimates of the position of 25 2D points of the human body and 21 2D points of the hand. OpenPose was used for motions including FT, HM, TT, LA, AFC, GAIT, and POS. Since some estimation of motions by OpenPose related to joints could cause inaccurate result when asking patients to straighten their arms (including PSOH, PTOH, and KTOH). For alleviating this impact, instead of OpenPose, we used HRNet (Wang et al., 2020) to increasing the accuracy during joint estimation.

In regard to the relationships between motions and dependent variables, features for the Rig-UE model were from seven motions related to the upper extremities, including FT, HM, PSOH, AFC, PTOH, KTOH, and GAIT. Features for the Rig-LE model were from four motions related to the lower extremities, including LA, AFC, and GAIT. In addition to the features used in the Rig-LE model, the PS model included one additional POS motion, i.e., the standing posture, which can represent the balance of the participant while remaining still. For the Rig-Neck model, we used all the motions.

Parkinson’s disease impacts the left and right limb of patients. Especially for PD of early stage, these patients can have significantly severe limb side, which means directly using feature of left or right can cause problem of inconsistency. To remove the impact of the severe limb side, we calculated some parameters using the two sides of the extremities to represent the overall condition or calculated the difference between the two sides of the participant. For a specific feature related to the two sides, for example, the release speed during FT, we calculated the mean, maximum, and minimum values for both hands and the absolute difference between right hand and left hand.

### 2.5. Feature extraction

**Figure 2:**
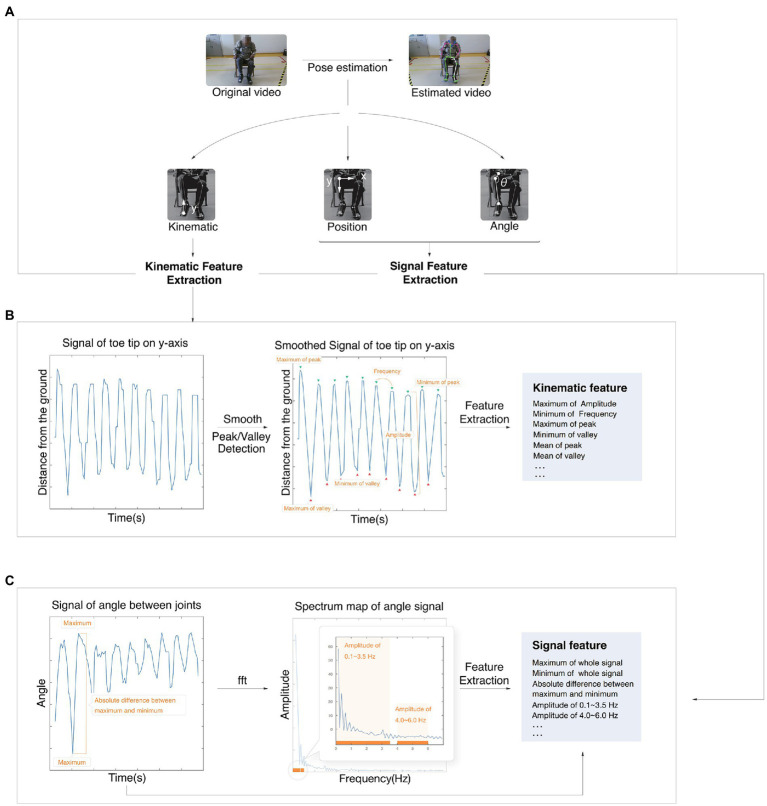
Process of the feature calculation. **(A)** The process from original video to estimated video. **(B)** Example of a kinematic feature calculation. **(C)** Example of signal feature calculation. fft, fast Fourier transform algorithm.

For each motion signal except for facial expression, there were three kinds of basics, including kinematic basics, position basics, and angle basics (shown in Figure 2A). Kinematic features reflected the performance of this motion, including speed, amplitude, hesitation, and decrement. The position feature and angle feature reflected the position of the joint or angle of range of motion during the test, respectively. The following details of feature calculation are described using the motion of LA as an example. The whole motion is presented in relation to the *y*-axis position of the toe tip. The original signal was then smoothed by smooth spline and detected to find the positions of peaks and valleys.

Kinematic features, including the maximum amplitude and minimum frequency, were extracted based on peaks and valleys (shown in Figure 2B). Both position basics and angle basics were performed by a signal extraction algorithm.

The position basics were based on both sides of the joint, and the figure shows the joint of the knee as an example. The angle basics were based on the angle between joints, and the figure shows the ROM of the knee as an example (Figure 2C). Some time-domain features, such as the maximum, were extracted directly from the original signal.

Other frequency-domain features were analyzed by the fast Fourier transform algorithm (FFT) and then extracted. Other features were the slope of the linear line based on peaks, quantile, standard deviation, root mean square, absolute mean, kurtosis coefficient, skewness coefficient, and so on.

### 2.6. Machine learning approach

Light gradient boosting machine (LightGBM; Ke et al., 2017) is a novel gradient-based decision tree model that can deal with a large number of features and output information gain for feature selection. Gains from the LightGBM were used to perform feature selection before the validation process. Leave-one-out cross validation (LOOCV) was performed to evaluate the performance of each model with different numbers of features, and then the model with the highest accuracy and weighted kappa (*k*) values was selected as the final full model. Then, the model was performed with the data from the test set to evaluate the performance of our models. Highest predicted proportion of score class was selected as the predicted score.

Three kinds of parameters were used to estimate the performance of our models. To estimate the accuracy of our models, two parameters were used. Absolute accuracy (*ACC ± 0*) was the proportion of the number for which the difference between the predicted score and true label was equal to zero. Acceptable accuracy (*ACC ± 1*) was the proportion of the number for which the difference between the predicted score and true label was less than 1. To estimate the consistency of our models, *k* was used. Six discrete levels were used to interpret the performance: <0.00, Poor; 0.00–0.20, Slight; 0.21–0.40, Fair; 0.41–0.60, Moderate; 0.61–0.80, Substantial; and 0.81–1.00, Almost Perfect (Landis and Koch, 1977). To estimate the relationship between the true label and predicted score, Spearman’s correlation coefficient (*rho*) was used. Five discrete levels were used to interpret the performance: 0.00–0.10, Negligible; 0.10–0.39, Weak; 0.40–0.69, Moderate; 0.70–0.89, Strong; and 0.90–1.00 Very Strong (Schober et al., 2018).

### 2.7. Item minimization and feature interpretation

To reduce the stress of patients, our study included an item minimization process. Based on the previous final full model, features from each item were removed from the total full feature set. Then, each new feature set was trained by a previous machine learning approach to find the feature set with the best performance. This feature set was established as the initial feature set for the next round, and this process continued until the last item. This procedure results in a feature set with acceptable performance and fewer required motions.

## 3. Results

### 3.1. Dataset of this study

As shown in Table 1, there were 89 patients (63.29 ± 10.26 years old) in train set and 15 patients (60.66 ± 11.47 years old) in test set. The mean ± SD of disease duration of train set and test set were 6.17 ± 5.03 and 5.84 ± 3.63 years. The demographic values including age, disease duration, and sex between train and test set were matched (value of *p* > 0.05). The other variables including total MDS-UPDRSIII score, rigidity total score, and postural stability were also matched.

**Table 1:** Demographic data of this study.

	Train set (*N* = 89)	Test set (*N* = 15)	Value of *p*
Sex, female^#^	39(43.8%)	5(33.3%)	0.6326
Age, year^&^	63.29 ± 10.26	60.66 ± 11.47	0.4149
Disease duration, year^&^	6.17 ± 5.03	5.84 ± 3.63	0.7567
MDS-UPDRSIII total score^&^	35.40 ± 16.26	36.40 ± 14.63	0.8129
Rigidity total score^&^	8.36 ± 3.61	9.2 ± 3.76	0.4172
Postural stability^†^	1[0,2]	1[0,1]	0.1762

Categorical values are represented as number and percentage; Normally distributed values are represented as mean ± SD; Non-normally distributed values are represented as median (25% quantile, 75% quantile). ^#^Based on Chi-square test; ^&^Based on T-test; ^†^Based on Wilcoxon test. Movement Disorder Society’s Unified Parkinson’s Disease Rating Scale Part III.

### 3.2. Performance when using all motions

After feature selection and outputting the final model, the number of motions used in the three models, with the exception of Rig-Neck, was the same as in our initial design. The number of motions used for Rig-Neck decreased from 10 to 8. As shown in Table 2, values of *ACC ± 0* of all four models were greater than 0.70. The absolute accuracies of the three models, including Rig-UE, Rig-Neck, and PS, were 0.73, while the value of Rig-LE was 0.70. All values of *ACC ± 1* were greater than 0.85. The Rig-LE and Rig-UE models had the highest value (0.97), followed by PS (0.93). Rig-Neck (0.87) had a value lower than 0.90. The correlation coefficients between predicted scores and true labels were greater than 0.60, and all of these coefficients were significant with *p* values lower than 0.05. The model with the highest correlation coefficient (0.76, Strong) was Rig-LE. The three values for Rig-UE, Rig-Neck, and Rig-PS were 0.64 (Moderate), 0.60 (Moderate), and 0.68 (Moderate), respectively. All of the *k* coefficients were greater than 0.50, and the models with the *k* were Rig-LE (0.66, Substantial) and PS (0.66, Substantial), followed by Rig-Neck (0.60, Moderate) and Rig-UE (0.58, Moderate). To summarize, the Rig-LE model had the highest consistency and correlation, and the Rig-Neck model had a relatively lower correlation and consistency and *ACC ± 1*.

**Table 2:** Performance of four models considering all motions.

	Rig-UE	Rig-LE	Rig-Neck	PS
rho	0.64	0.76	0.60	0.68
rho, value of p	<0.01	<0.01	0.02	0.01
Weighted kappa (CI)	0.58 (0.34–0.81)	0.66 (0.47–0.86)	0.60 (0.27–0.94)	0.66 (0.34–0.98)
ACC ± 0	0.73	0.70	0.73	0.73
ACC ± 1	0.97	0.97	0.87	0.93
Feature number	35	30	25	25
Motion list	KTOH, AFC, GAIT, PTOH, FT, PSOH, HM	AFC, GAIT, TT, LA	TT, AFC, FT, FE, GAIT, PTOH, LA, PSOH	AFC, POS, GAIT, TT, LA
Number of motions	7	4	8	5

CI, confidence interval; Rig, rigidity; UE, upper extremity; LE, lower extremity; PS, postural stability; AFC: arising from chair; FE, facial expression; FT, finger tapping; HM, hand movements; KTOH, kinetic tremor of the hands; LA, leg agility; POS, posture; PSOH, pronation/supination of the hands; PTOH, postural tremor of the hands; TT, toe tapping; AFC: arising from chair; and FE, facial expression.

### 3.3. Performance after item minimizing

After minimizing indirect motions, we attempted to retain models with *ACC ± 0* greater than 0.70. However, only the Rig-Neck and Rig-UE models achieved this goal. As shown in Table 3, the values of *ACC ± 0* of the Rig-Neck and Rig-UE models were 0.73 and 0.70, respectively, while the highest values of Rig-LE and PS after item minimization were 0.63 and 0.67, respectively. Therefore, we consider only the Rig-Neck and Rig-UE models as meaningful results. With regard to the consistency of performance, the *k* values of the Rig-Neck and Rig-UE models were 0.67 (Substantial) and 0.54 (Moderate), respectively. The correlations were 0.71 (Substantial) and 0.59 (Moderate). The final motions after the item minimization process for Rig-Neck were FT, PSOH, GAIT, and TT. For Rig-UE, the final motions were KTOH, GAIT, PTOH, FT, HM, and PSOH.

**Table 3:** The performance of four models after item minimization.

	Rig-UE	Rig-LE	Rig-Neck	PS
rho	0.59	0.49	0.72	0.69
rho, value of *p*	0.00	0.01	0.00	0.00
Weighted kappa (CI)	0.54 (0.28–0.79)	0.45 (0.18–0.72)	0.67 (0.38–0.96)	0.65 (0.34–0.95)
ACC ± 0	0.70	0.63	0.73	0.67
ACC ± 1	0.97	0.87	0.93	0.93
Feature number	20	30	45	45
Motion list	KTOH, GAIT, PTOH, FT, HM, PSOH	GAIT, TT, LA	FT, PSOH, GAIT, TT	POS, GAIT, LA
Number of motions	6	3	4	3

CI, confidence interval; Rig, rigidity; UE, upper extremity; LE, lower extremity; PS, postural stability; AFC, arising from chair; FE, facial expression; FT, finger tapping; HM, hand movements; KTOH, kinetic tremor of the hands; LA, leg agility; POS, posture; PSOH, pronation/supination of the hands; PTOH, postural tremor of the hands; TT, toe tapping; AFC: arising from chair; and FE, facial expression.

### 3.4. Feature analysis

**Figure 3:**
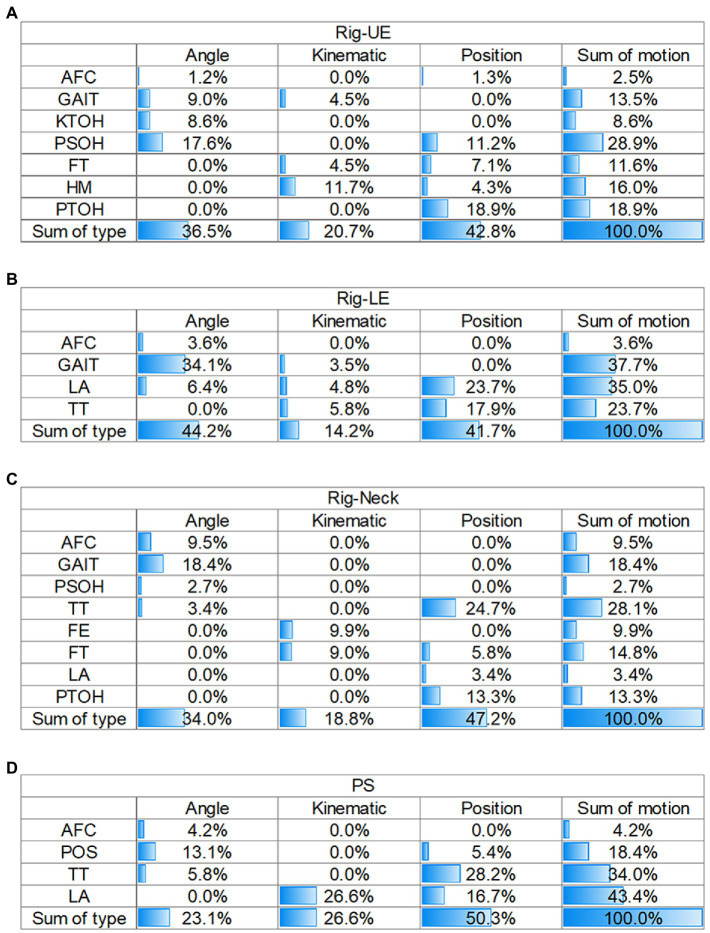
Matrix of contributions of different sources in the four models. **(A)** Rig-UE. **(B)** Rig-LE; **(C)** Rig-Neck. **(D)** PS. Rig, rigidity; UE, upper extremity; LE, lower extremity; PS, postural stability; AFC: arising from chair; FE, facial expression; FT, finger tapping; HM, hand movements; KTOH, kinetic tremor of the hands; LA, leg agility; POS, posture; PSOH, pronation/supination of the hands; PTOH, postural tremor of the hands; TT, toe tapping; AFC: arising from chair; and FE, facial expression.

The matrix of the sum of GAIN, based on motion and feature type, was used to interpret the contribution of each model (as shown in Figure 3).

For the Rig-UE model and the motions (Figure 3A), the highest contributing motion was PSOH (28.9%), followed by PTOH (18.9%), HM (16.0%), and GAIT (13.5%). In regard to the type of feature, position (42.8%) was the most important, followed by angle (36.5%), and the kinematics was the least important contributor.

For the Rig-LE model and the motions (Figure 3B), the two highest contributors were GAIT (37.7%) and LA (35.0%). TT (23.7%) also had a relatively high contribution compared to AFC (3.6%). In regard to the dimension of feature type, the values of angles (44.2%) and positions (41.7%) were close, while the kinematics (14.2%) was relatively low.

For the Rig-Neck model (Figure 3C), the four motions with a cumulative gain higher than 10% were TT (28.1%), GAIT (18.4%), FT (14.8%), and PTOH (13.3%). Regarding the dimension of feature type, position (47.2%) contributed the most, followed by angle (23.1%).

For the PS model (Figure 3D), the top three motions with the highest importance were LA (33.8%), GAIT (31.7%), and TT (23.4%), while AFC (2.1%) and POS (9.1%) contributed less. On the dimension of feature type, position (43.5%) and angle (41.5%) contributed at nearly the same level, while the kinematics (15.1%) type contributed less.

By comparing the results of four matrices on the dimension of motion, the importance of GAIT was always higher than 10%, while the value of the AFC was always at a relatively low level (<10%). In addition, TT had a high importance greater than 20.0% in the Rig-LE, Rig-Neck, and PS models. For the dimension of feature type, position and angle were the two primary types with a level of importance greater than 35%. Compared with other models, the Rig-UE model had the highest importance of the kinematic features.

## 4. Discussion

From the results of the feature analysis, we found that the features of angle and position were more important than the kinematics feature, which indicated that during the whole process of motion, the performance during muscle and joint stability can contribute more to our models than the kinematic features, including speed and amplitude. In addition, some motions remained important in both the whole model and the minimized model, and the GAIT motion was always present. During the analysis of features, we found that features from GAIT can contribute greatly (greater than 13%) in all four models. Gait impairment was regard as a significant characteristic of PD progression (Nutt et al., 2011), and serval studies had found that information of walking was correlated with rigidity or PS. Wright et al. (2007) suggested that sum of rigidity scores in UPDRS was correlated with hip torque during walking (*r* = 0.73, *p* < 0.001). Schaafsma et al. (2003) suggested that PS was correlated with the coefficient of variation of stride length during walking (*r* = 0.50, *p* = 0.003). These results and our findings indicated features from walking can be representative not only for the overall motor condition of PD, but also for rigidity and PS.

Several studies related to remote vision assessment, removed rigidity, and postural stability since they were hard to achieve in the remote condition (Stillerova et al., 2016; Xu et al., 2021). Instead of directly touching and using pullback test, in our study, by using a machine learning system based on RGB camera and clinical features extracted from the MDS-UPDRS III, we developed four models for estimating rigidity and PS in relation to PD, achieving an accuracy greater than 70%. The pattern (without touching and vision-based) of assessment in our study can be workable in remote circumstance.

For assessing rigidity, previous studies used objective quantitative methods, such as servomotors, inertial sensors, and biomechanical and neurophysiological studies of muscles, rigidity can be quantitatively assessed with good validity and reliability (Cano-de-la-Cuerda et al., 2011). However, these assessment need examiners touching and placing sensors on the muscle of PD patients, which can be relatively less appropriate compare with vision-based methods. Some studies tried to connect rigidity to motion completed on smartphone or electronic device. The study of Trager et.al found that finger tapping speed captured by an engineered keyboard was correlated with upper extremity (*r* = 0.58, *p* < 0.0001; Trager et al., 2020). Team of Wilkins et al. (2022) found that release slope during finger tapping in a portable quantitative digitography device was correlated with rigidity sub-score (*r* = −0.43, *p* < 0.0001). These studies proved rigidity can be correlated with performance of other motions, but they did not apply them to build evaluation model for clinical score. We managed to evaluated rigidity by other motions by simple vision algorithm.

For assessing PS, the original rule of MDS-UPDRSIII is performing pullback testing on the participant by a trained examiner for ensuring the safety. Yang et al. (2022) used vision-based method and deep learning to build the model for assessing PS during the pullback test and achieved excellent precision. However, this test cannot be unsafe to be done in remote circumstance since it is unlikely that all caregivers are qualified. Serval studies also tried to use sensors of smartphone and find features associated with PS, or assess PS remotely by other motions such as turning, walking and quiet stance. The study of Borzì et al. extracted features from a smartphone placed on the waist of participant during 180° turning, and found the probability from a binary model based on these features for discriminating mild PS condition and severe PS condition, was correlated with PS score (*r* = 0.73, *p* < 0.0001; Borzì et al., 2020b). The other study of Borzì et.al extracted features from a waist-mounted smartphone during quiet stance of 30 s and built binary model for differentiating mild and severe postural instability (Borzì et al., 2020a). By analyzing other motions, these studies found that PS can be correlated and evaluated by other motions based on sensors of smartphones. We tried the other way of using vision, which can be relatively easier for rechecking the history about the detailed during the motion for making sure the quality of motions.

Although several previous studies have indicated that rigidity or PS is related to other motions or using sensors of smartphone to evaluate PS, to our knowledge, our study is the first to develop a system for evaluating rigidity and PS scores indirectly by other motions and the RGB algorithm. Compared with correlation analysis, model building can perform and be applied in real-world conditions. Since the evaluation of rigidity or PS has been a problem for remote follow-up, our study can be an alternative program for completing all the items of the MDS-UPDRS III so that the total score can be summed to represent the clinical situation of patients with PD. In addition, in conditions such as the COVID-19 environment, social distancing between patients and neurologists is maintained to reduce the infection rate. This system can be more meaningful under such circumstances.

Another meaningful aspect of this study is the clinical interpretation between indirect motions and rigidity or PS. Our study summarized this relationship by using the matrix of feature types and motions. This could provide a foundation for subsequent studies to choose specific motions that would be the most worthwhile to represent the severity of rigidity or PS. To capture the importance of this, we performed item minimizing, which allowed us to show how the lowest number of motions can result in an acceptable model.

There are serval limitations in our study. Our study was single-centered, and the group that obtained scores of four were merged into the group that scored three because of the lack of samples having a score of four, and the sample size was relatively limited. We are now preparing for a multicenter study to provide a more solid validation of this system. During this work, more patients with scores of four will be considered. Our system now has completed the model with the five items in the MDS-UPDRS III. Models for the remaining items will be completed in the future. Since the basic frame of our study is RGB, which can be workable in many smartphones, it is possible to consider that the whole MDS-UPDRS III evaluation system can be applied on smartphones so that patients with PD can complete the evaluation at home independently or with the help of their caregivers. If this system for evaluating the whole MDS-UPDRS III is accomplished, neurologists can better and more efficiently track the condition of their patients using these remote artificial intelligence patterns.

## Data availability statement

The raw data supporting the conclusions of this article will be made available by the authors, without undue reservation.

## Ethics statement

The studies involving human participants were reviewed and approved by the ethics committees of Beijing Tiantan Hospital; reference ID: KYSQ 2021–080-01. The patients/participants provided their written informed consent to participate in this study.

## Author contributions

L-YM, Z-LC, and YL participated in the design of the study and drafted the manuscript. W-KS and CC analyzed data and contributed to the manuscript. TF and KR carried out the conceptualization of the study and reviewing and critiquing the article at the same time. ZW, X-MW, and LC collected medical data of the patients. All authors contributed to the article and approved the submitted version.

## Funding

This research was supported by Natural Science Foundation of China (Nos. 82071422 and 81571226), Beijing Natural Science Foundation (Nos. 7212931 and 7164254), National Keypoint Research and Invention Program of the Thirteenth (2016YFC1306501), Capital Characteristic Clinic Project (Z171100001017041), and Beijing Municipal Science and Technology Commission (Nos. Z151100003915117 and Z151100003915150).

## Conflict of interest

The authors W-KS, CC, KR, Z-LC, and YL with Gyenno Science Co., Ltd. affiliations are current Gyenno Science employees.

The remaining authors declare that the research was conducted in the absence of any commercial or financial relationships that could be construed as a potential conflict of interest.

The handling editor JM declared a shared parent affiliation with the authors TF, L-YM, ZW, X-MW, and J-NJ at the time of review.

## Publisher’s note

All claims expressed in this article are solely those of the authors and do not necessarily represent those of their affiliated organizations, or those of the publisher, the editors and the reviewers. Any product that may be evaluated in this article, or claim that may be made by its manufacturer, is not guaranteed or endorsed by the publisher.
